# Development of Loop-Mediated Isothermal Amplification with a Lateral Flow Dipstick for Detection of the *Bla*_OXA-23-like_ Gene from Carbapenem-Resistant *Acinetobacter baumannii*

**DOI:** 10.3390/ijms27177761

**Published:** 2026-08-29

**Authors:** Saranthum Phurijaruyangkun, Pongbun Tangjitrungrot, Kantima Choosang, Adun Bunchaleamchai, Naiyana Wattanakul, Bajaree Jantrapanukorn, Rungnapa Veeramano, Suphitcha Augkarawaritsawong, Khurawan Kumkrong, Supatra Areekit, Kosum Chansiri, Somchai Santiwatanakul, Pornpun Jaratsing, Sawanya Pongparit

**Affiliations:** 1 Faculty of Medical Technology, Rangsit University, Pathum Thani 12000, Thailand; saranthum.p@rsu.ac.th (S.P.); kantima.c@rsu.ac.th (K.C.); adun.b@rsu.ac.th (A.B.); bajaree.j@rsu.ac.th (B.J.); rungnapa.v@rsu.ac.th (R.V.); suphitcha.a@rsu.ac.th (S.A.); khurawan.k@rsu.ac.th (K.K.); 2 Center of Excellence in Biosensors, Panyananthaphikkhu Chonprathan Medical Center, Srinakharinwirot University, Nonthaburi 11120, Thailand; pongbun@g.swu.ac.th (P.T.); supatraa@g.swu.ac.th (S.A.); titi41@yahoo.com (S.S.); 3 Nopparat Rajathanee Hospital, Bangkok 10230, Thailand; naiya447@gmail.com; 4 Innovative Learning Center, Srinakharinwirot University, Bangkok 10110, Thailand; 5 Department of Biochemistry, Faculty of Medicine, Srinakharinwirot University, Bangkok 10110, Thailand; kosum@g.swu.ac.th

**Keywords:** carbapenem-resistant *Acinetobacter baumannii*, loop-mediated isothermal amplification, lateral flow dipstick, *bla*_OXA-23-like_ gene

## Abstract

Carbapenem-resistant *Acinetobacter baumannii* (CRAB) is a major cause of hospital-acquired infections. Conventional phenotyping for carbapenem resistance requires more than 24 h, which delays clinical decisions and increases the risk of outbreaks. This study aims to develop a lateral flow dipstick (LFD) assay that detects loop-mediated isothermal amplification (LAMP) products targeting the *bla*_OXA-23-like_ gene, providing results within 1–1.5 h. Primer specificity was evaluated using seven Gram-positive and nine Gram-negative species commonly present in clinical samples, as well as carbapenem-susceptible *A. baumannii* and CRAB isolates harboring other resistance genes. The assay detected the *bla*_OXA-23-like_ gene at concentrations as low as 1.57 pg/µL, demonstrating ~10-fold greater sensitivity than conventional PCR. The gene was present in 224/268 CRAB samples (83.6%) and absent in 100 carbapenem-susceptible *A. baumannii* samples. Results were fully concordant with PCR, yielding 100% sensitivity and specificity. The method requires no expensive or complex instrumentation. Future studies should validate the assay across multiple hospitals and clinical specimens to confirm its generalizability and clinical utility. Multiplex LAMP with dual LFD readouts could also enable simultaneous detection of additional carbapenem resistance genes, supporting rapid CRAB surveillance and outbreak control.

## 1. Introduction

Antimicrobial resistance (AMR) is a major global public health threat, contributing to prolonged hospitalization, increased mortality, and rising healthcare costs, driven largely by inappropriate antimicrobial use and inadequate infection prevention [1,2]. The WHO has classified carbapenem-resistant *Acinetobacter baumannii* (CRAB) as a critical-priority pathogen due to its multidrug resistance and limited treatment options [3].

CRAB causes serious healthcare-associated infections, including ventilator-associated pneumonia, bloodstream, urinary tract, meningitis, and surgical-site infections, particularly in critically ill and immunocompromised patients [4].

*Acinetobacter baumannii* is a Gram-negative coccobacillus that colonizes humans and hospital environments, forms biofilms on surfaces and medical devices, and causes severe healthcare-associated infections. Its high antimicrobial resistance limits treatment options, with mortality rates reaching 70% [5,6,7,8].

Carbapenems are broad-spectrum, bactericidal β-lactams that inhibit bacterial cell-wall synthesis and are widely used against multidrug-resistant Gram-negative infections [9]. However, excessive carbapenem use promotes resistance, underscoring the need for antimicrobial stewardship and judicious use [10].

CRAB resistance is driven mainly by class D carbapenemases (CHDLs), particularly *bla*_OXA-23-like_, the predominant global carbapenemase. Other CHDLs include *bla*_OXA-24/40-like_ and *bla*_OXA-51-like_, the latter often associated with IS*Aba1*. Metallo-β-lactamases (NDM, IMP, and VIM) are less common (<3% of isolates) [11]. The *bla*_OXA-23-like_ gene is commonly carried on chromosomes or plasmids within Tn2006–Tn2009, associated with IS*Aba1* or IS*Aba4*, which enhance gene expression and facilitate carbapenem resistance dissemination [12]. 

The *bla*_OXA-23-like_ gene is the predominant acquired carbapenemase gene in CRAB worldwide, with geographic variation; a global study of 313 isolates from 47 countries detected acquired OXA-type carbapenemases in 96% of isolates [13].

The 2024 annual report of NARST showed that, in Thailand, *Acinetobacter* spp. had the highest imipenem resistance (~75%), far exceeding that of *Pseudomonas aeruginosa* (20.3%), *Klebsiella pneumoniae* (11.9%), and *Enterobacter cloacae* (7.3%) [14].

In Thailand, *bla*_OXA-23-like_ is the predominant class D carbapenemase gene in CRAB, detected in 82.6% of isolates in 2013–2015 and 68.3% in 2016–2017. Our previous study found that CRAB prevalence increased from 68% in 2017 to 77.7% in 2018, with 80.6% of isolates carrying *bla*_OXA-23-like_ and no metallo-β-lactamase genes detected [15,16,17].

Most CRAB isolates are MDR or XDR, leaving limited treatment options. Colistin remains a last-line agent but is limited by nephrotoxicity, while newer agents such as sulbactam-durlobactam, cefiderocol, and tigecycline, along with combination therapy, offer additional options for severe infections [18].

Rapid CRAB detection enables timely treatment and infection control. Although PCR rapidly detects carbapenem resistance genes, it requires specialized equipment and trained personnel, limiting its use in resource-limited settings [19].

LAMP is a rapid, cost-effective amplification method that can amplify target DNA up to 10^9^-fold within 1 h at 60–65 °C using simple equipment, making it suitable for point-of-care and resource-limited settings [20,21]. Although LAMP products can be confirmed by gel electrophoresis, this method is time-consuming, requires specialized equipment, and may not reliably distinguish specific from nonspecific amplification based on the characteristic ladder-like pattern [22].

Alternative detection methods include SYBR Green fluorescence, which detects LAMP products by binding double-stranded DNA [23]. Hydroxynaphthol blue (HNB) enables visual LAMP detection through a violet-to-sky-blue color change [24,25]. Alternative detection methods include pH-sensitive dyes, such as phenol red, cresol red, and neutral red, which change color during LAMP amplification [22]. Gold nanoparticles (AuNPs) enable colorimetric DNA detection through probe-mediated aggregation changes but are sensitive to ionic strength, pH, and temperature, which can cause nonspecific aggregation and ambiguous results [26,27].

Although LAMP is highly sensitive, it can produce false positives due to poor primer design, primer-dimer formation, and nonspecific amplification. Conventional detection methods generally detect amplification products rather than target-specific sequences, increasing the risk of false positives [23].

To overcome these limitations, Milenia Biotec GmbH developed LAMP–lateral flow dipstick (LAMP-LFD), which combines isothermal amplification with sequence-specific probe detection, enabling visual results within 60 min without a thermal cycler while maintaining high sensitivity and specificity [28]. However, analytical specificity depends on the sequence-specific design of both the primers and probe.

LAMP assays can achieve 10–1000-fold greater analytical sensitivity than conventional PCR, with improvements reported for *Plasmodium falciparum* (10-fold), *Helicobacter pylori* (100-fold), and *Mycoplasma ovipneumoniae* (up to 1000-fold), while maintaining high concordance with reference methods [21,29,30].

LAMP assays can rapidly detect carbapenemase genes, with up to 1000-fold greater sensitivity than PCR in some *Enterobacterales* applications. For CRAB, reported LAMP assays achieve 88.6–100% sensitivity with rapid turnaround times [31]. A recent fluorescent lateral flow assay targeting *bla*_OXA-23-like_ delivered results within 43 min and was up to 28-fold more sensitive than real-time PCR but required a fluorescence reader [32].

This study developed a LAMP-LFD assay targeting *bla*_OXA-23-like_ and evaluated its sensitivity, specificity, detection limit, and assay time against conventional PCR for CRAB detection.

## 2. Results

### 2.1. Optimization of the PCR Assay Annealing Temperature

PCR annealing temperatures of 42–55 °C were evaluated (Supplementary Figure S1). Specific amplification of the 195-bp *bla*_OXA-23-like_ amplicon occurred at 50–55 °C without nonspecific bands; therefore, 50 °C was selected for subsequent experiments.

### 2.2. Optimization of the PCR Assay’s Magnesium Chloride Concentration

Optimization of magnesium chloride (MgCl_2_) concentration (2–6 mM) showed specific amplification of the 195-bp *bla*_OXA-23-like_ gene target at 3–5.5 mM without nonspecific bands; therefore, 3 mM MgCl_2_ was used for PCR. (Supplementary Figure S2).

### 2.3. The Optimization of the Amplification Temperature of the LAMP Assay

The LAMP amplification temperature was optimized from 50 to 70 °C (Supplementary Figure S3). Clear ladder-like bands were observed at 62.1 to 64.8 °C; therefore, 63 °C was selected as the optimal amplification temperature for detecting the *bla*_OXA-23-like_ gene.

### 2.4. Optimization of the LAMP Assay’s Magnesium Sulfate Concentration

MgSO_4_ concentrations of 2.4–6.0 mM were evaluated at 63 °C (Supplementary Figure S4). Intense ladder-like bands were observed at 4.8–6.0 mM, with 5.2 mM selected as optimal for the LAMP assay.

### 2.5. Optimization of the LAMP Assay’s Amplification Time

LAMP amplification times of 15–60 min were evaluated (Supplementary Figure S5). Clear ladder-like bands appeared at 45 and 60 min; therefore, 45 min was selected as the optimal amplification time.

### 2.6. Determining the Primer Specificity

**Figure 1:**
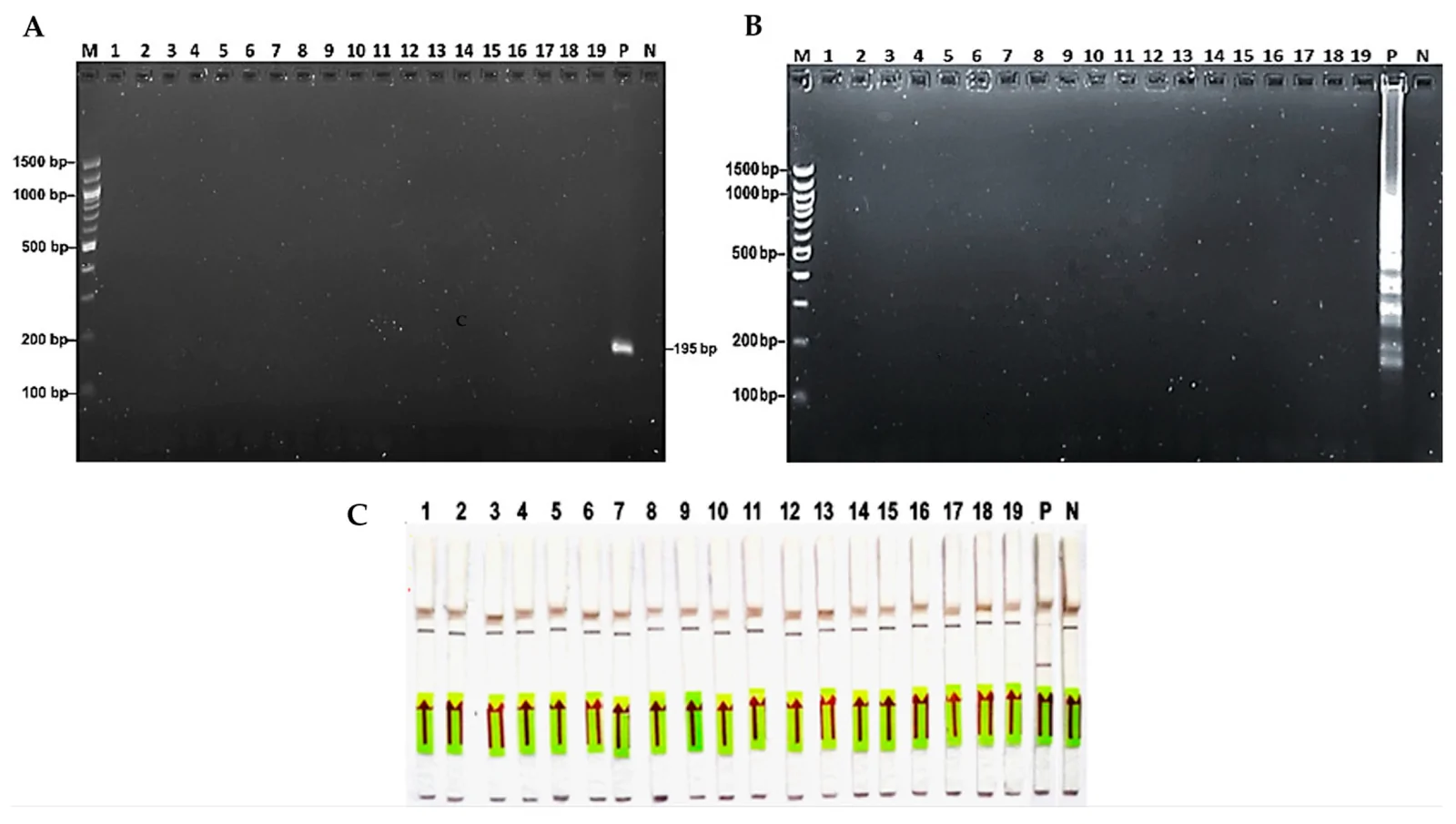
Specificity of (**A**) PCR, (**B**) LAMP, and (**C**) LAMP-LFD assays for detecting the *bla*_OXA-23-like_ gene. Lane/strip M: 100 bp Plus DNA Ladder; P: positive control (*A. baumannii* AB029); N: negative control (Milli-Q water). Lanes/strips 1–7: *Staphylococcus aureus* ATCC^®^ 25923, oxacillin-resistant *S. aureus* ATCC^®^ 43300, D-test-positive *S. aureus*, *Enterococcus faecalis* ATCC^®^ 29212, vancomycin-resistant *E. faecalis* ATCC^®^ 51299, vancomycin-resistant *E. faecium* ATCC^®^ 700221, and *Streptococcus pneumoniae* ATCC^®^ 49619. Lanes/strips 8–16: *A. lwoffii* ATCC^®^ 15309, *Escherichia coli* ATCC^®^ 25922, carbapenem-resistant *K. pneumoniae* ATCC^®^ BAA-1705, ESBL-producing *K. quasipneumoniae* ATCC^®^ 700603, *K. aerogenes* ATCC^®^ 13048, *Enterobacter cloacae* ATCC^®^ 23355, *Pseudomonas aeruginosa* ATCC^®^ 27853, *P. fluorescens* ATCC^®^ 13525, and *Burkholderia cepacia* ATCC^®^ 25416. Lanes/strips 17–19: carbapenem-susceptible *A. baumannii* ATCC^®^ 19606, CRAB isolate with *bla*_OXA-58-like_, and CRAB isolate with *bla*_OXA-24/40-like_.

The specificity of the PCR, LAMP, and LAMP-LFD primer sets targeting *bla*_OXA-23-like_ was confirmed against 16 bacterial species, with no cross-reactivity observed (Figure 1). 

### 2.7. Determining the Analytical Sensitivity

**Figure 2:**
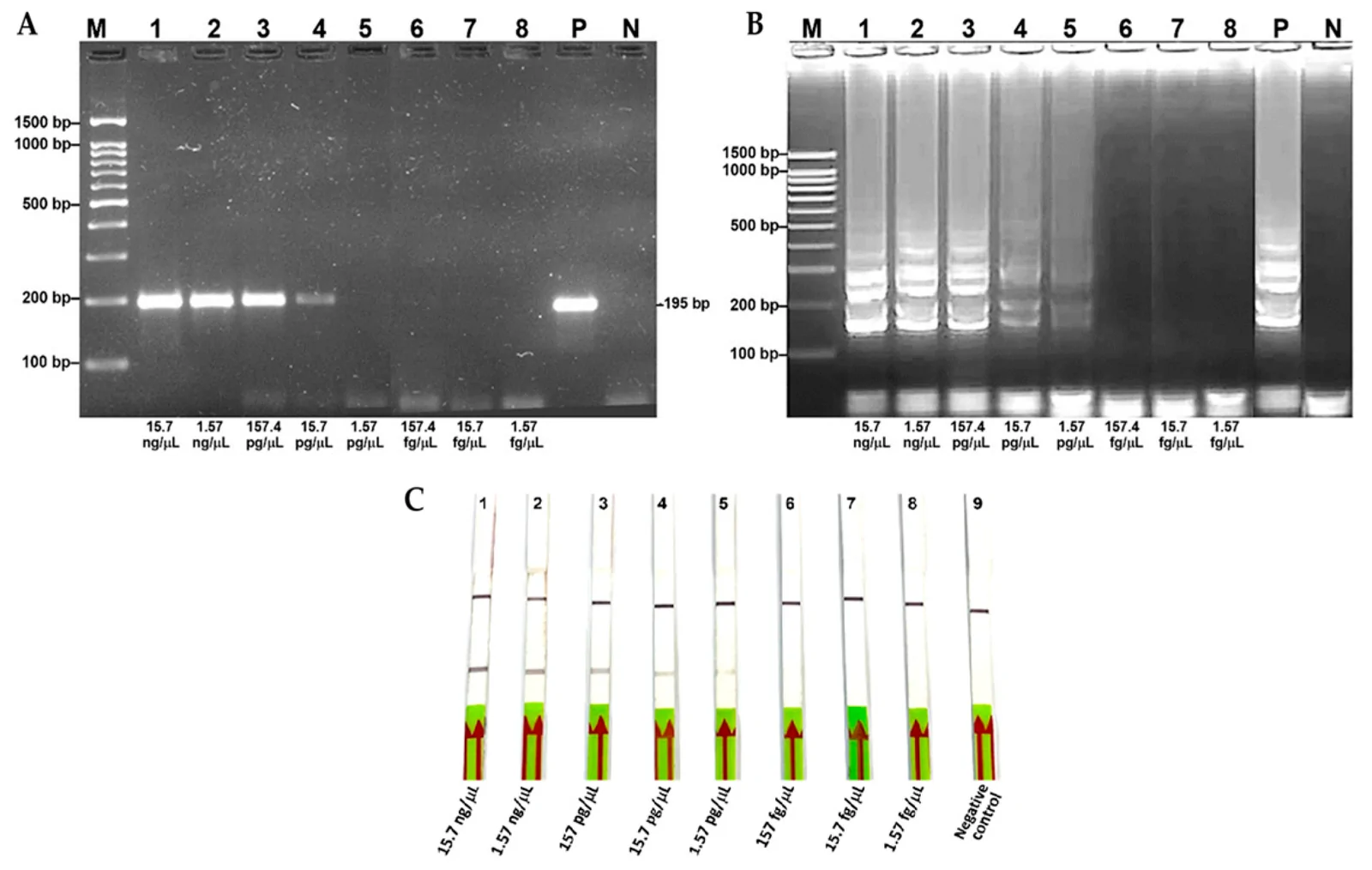
Analytical sensitivity of PCR, LAMP, and LAMP-LFD for detection of the *bla*_OXA-23-like_ gene. (**A**) PCR: LOD = 15.7 pg/µL. (**B**) LAMP: LOD = 1.57 pg/µL. (**C**) LAMP-LFD: LOD = 1.57 pg/µL, representing a ~10-fold improvement in analytical sensitivity over PCR.

Analytical sensitivity or limit of detection (LOD) was assessed using serially diluted *A. baumannii* AB029 DNA. The LAMP and LAMP-LFD assays detected the *bla*_OXA-23-like_ gene at concentrations as low as 1.57 pg/µL, representing 10-fold greater sensitivity than PCR (15.7 pg/µL), as shown in Figure 2.

### 2.8. Validity and Predictive Values

The performance of LAMP-LFD for detecting *bla*OXA-23-like was evaluated in 368 clinical *A. baumannii* isolates using PCR as the reference method. Among 268 CRAB isolates collected in 2018–2023, 224 (83.6%) were PCR-positive, and 44 (16.4%) were negative, while none of the 100 carbapenem-susceptible isolates carried *bla*OXA-23-like. LAMP-LFD, LAMP, and PCR showed complete concordance (Table 1; Supplementary Figures S6–S10). Compared with PCR, LAMP-LFD demonstrated 100% sensitivity, specificity, accuracy, PPV, and NPV, with perfect agreement (κ = 1; LR+ = ∞; LR− = 0). The 95% CI for the *bla*OXA-23-like-positive proportion among CRAB isolates was 79.1–88.0% (Supplementary Table S2). The assay enabled rapid visual detection of *bla*OXA-23-like within 1.5 hours.

## 3. Discussion

Carbapenem resistance in *A. baumannii* increased from 77.3% in 2018 to 79.6% in 2023, with all CRAB isolates exhibiting multidrug resistance and some classified as XDR or PDR. The LAMP-LFD assay showed no cross-reactivity with 16 non-target pathogens and achieved 100% sensitivity, specificity, and agreement with PCR among 368 isolates. It correctly detected all 224 *bla*_OXA-23-like_-positive isolates, including six co-harboring *bla*_OXA-58-like_, all 87 positive CRAB isolates from 2023, and all 100 carbapenem-susceptible isolates. Results were obtained within 1–1.5 h, including DNA extraction, using a dry-bath platform and lyophilized reagents, supporting its potential for rapid CRAB detection in resource-limited settings [33].

LAMP assays for detecting the *bla*_OXA-23-like_ gene have been reported. Yamamoto, et al. developed a LAMP method using a real-time turbidity assay for detecting *bla*_OXA-23_-containing CRAB in 120 sputum and rectal secretion samples from 108 patients. The LAMP method achieved a sensitivity of 100.0% with a limit of detection of 2 × 10^−3^ pg, which was 1000 times better than PCR; however, the method showed high sensitivity but required a real-time turbidimeter, making it not suitable for point-of-care testing [34]. Garciglia-Mercado et al. developed a SYBR Green I–based LAMP assay for carbapenem resistance genes in 33 environmental samples from an ICU outbreak. The LAMP assay detected the *bla*_OXA-23-like_ gene in CRAB at a higher rate (36.4%) than PCR (18.2%) and showed ten-fold greater sensitivity than PCR (1.0 ng/µL); the SYBR Green I–based LAMP assay detected any amplification product, not the specific target gene [35]. Kim et al. developed a LAMP agarose gel assay for carbapenem resistance genes in 75 CRAB from sputum samples. The LAMP assay detected the *bla*_OXA-23-like_ gene at a higher rate (72.0%) than PCR (69.3%) and showed tenfold greater sensitivity than PCR (10 pg/µL) [36].

Although several LAMP-based assays have been developed for carbapenemase gene detection, a LAMP-LFD assay targeting the predominant CRAB resistance determinant, the *bla*_OXA-23-like_ gene, has not been reported. This assay enables rapid visual detection without specialized equipment while maintaining high analytical sensitivity and diagnostic performance [37,38].

The LAMP-LFD assay provides a rapid, accurate, low-cost, and equipment-free method for detecting the *bla*_OXA-23-like_ gene in *A. baumannii,* making it well suited for point-of-care testing and resource-limited settings. Its rapid turnaround and high diagnostic performance could facilitate early identification of CRAB, support timely antimicrobial therapy, and strengthen infection prevention and surveillance.

However, LAMP’s rapid, high-yield amplification can increase the risk of carryover contamination and false-positive results. This limitation should be considered before performing LAMP, and it can be minimized by following standard contamination-control procedures, including physical separation of pre- and post-amplification areas and a unidirectional workflow from reagent preparation to sample addition area, amplification area, and lastly, the detection area. The dedicated pipettes in each area must be provided with aerosol-resistant filter tips. During sample addition, other reaction tubes should remain tightly closed, and aerosol generation should be avoided. Post-amplification tubes should never be opened in the reagent-preparation area. No-template controls should be included in each run, and work surfaces and equipment should be regularly decontaminated with appropriate disinfectants and UV irradiation [37].

Furthermore, prolonged LAMP amplification may increase nonspecific amplification and false-positive results. Therefore, extending the reaction time beyond the optimized duration, which is 45 to 60 min, may compromise assay specificity and prolonged LAMP amplification [38].

Moreover, this study was conducted at a single center; multicenter validation and evaluation using direct clinical specimens are needed. Future development of multiplex LAMP-LFD assays for simultaneous detection of multiple carbapenem resistance genes could further enhance antimicrobial resistance surveillance and outbreak control.

## 4. Materials and Methods

### 4.1. Bacterial Clinical Isolates

Groups 1 and 2 comprised non-duplicate clinical isolates of carbapenem-resistant *A. baumannii* (CRAB). Group 1 included 170 CRAB isolates collected in 2018, all carrying the *bla*_OXA-51-like_ gene; 137 also carried the *bla*_OXA-23-like_ gene, including six co-harboring the *bla*_OXA-58-like_ gene. Of the remaining 33 isolates, nine carried the *bla*_OXA-24/40-like_ gene, whereas 24 harbored the IS*Aba1*–*bla*_OXA-51-like_ gene. Group 2 included 98 CRAB isolates collected in 2023, and Group 3 comprised 100 carbapenem-susceptible *A. baumannii* isolates, with 50 collected in 2018 and 50 in 2023.

Clinical isolates were identified by MALDI-TOF MS (Beckman Coulter, Inc., Brea, CA, USA) and confirmed by biochemical testing. Antimicrobial susceptibility to imipenem, meropenem, and other agents was determined by broth microdilution using the Sensititre™ ARIS™ 2X system (Thermo Fisher Scientific, San Diego, CA, USA). Other bacterial species were included to evaluate primer specificity for PCR, LAMP, and LAMP-LFD assays. Ethical approval was obtained from the Rangsit University Ethics Committee (RSUERB2023-026). 

### 4.2. DNA Extraction

The tested isolates were cultured on blood agar at 35 °C for 24 h. Genomic DNA was extracted using the Presto Mini gDNA Bacterial Extraction Kit (Geneaid Biotech Ltd., New Taipei City, Taiwan, China) according to the manufacturer’s protocol. The procedure involved cell lysis with lysis buffer and proteinase K, silica membrane-based DNA purification, washing to remove the contaminants, and elution of purified DNA [39]. DNA was collected into two Eppendorf tubes; concentration and purity were measured using a NanoDrop™ 2000 spectrophotometer (Thermo Fisher Scientific, Waltham, MA, USA), and the DNA concentration was adjusted to 50 ng/µL. The DNA was stored at −20 °C for further use. One tube for screening, the other for confirmation.

### 4.3. Positive Control bla_OXA-23-like_

A carbapenem-resistant *A. baumannii* isolate AB029 carrying the *bla*_OXA-23_ gene served as the positive control, with 99% sequence identity to *A. baumannii* strain KKG1 with the *bla*_OXA-23_ gene (GenBank accession No. LC096086).

### 4.4. Primer Design for PCR and LAMP

Accurate primer design is essential for achieving high specificity and efficient amplification in PCR and LAMP assays, thereby reducing the risk of false-positive results [40]. The LAMP primer set, comprising FIP, BIP, F3, and B3 primers, targeted six regions of the *bla*_OXA-23_ gene from *A. baumannii* strain ARI-1 (GenBank accession No. AJ132105.1). This strain was used by many studies [41,42]. Primers were designed using PrimerExplorer V5 and validated by NCBI BLASTN; the set showing 100% identity and specificity among all *A. baumannii* strains was selected for further experiments. No nucleotide mismatches were observed within the binding regions of the essential inner primers (FIP and BIP), while only a limited number of single nucleotide variations were identified in the outer primer binding sites of a small number of sequences, and these were located outside the critical 3′ terminal regions.

**Figure 3:**
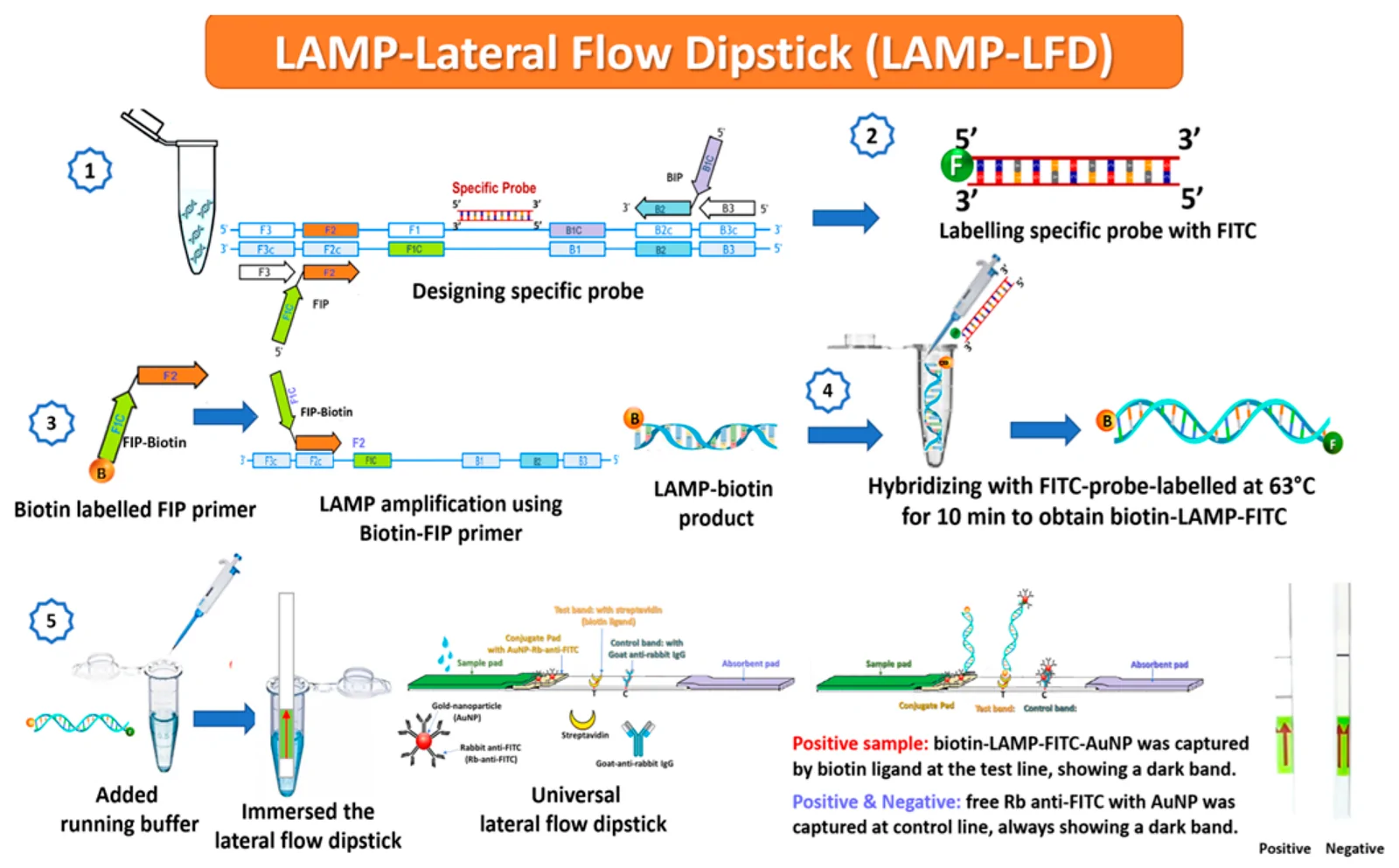
The LAMP-LFD assay was performed according to Milenia Biotec’s rapid lateral flow assay protocol and consisted of five steps: (**1**) probe design targeting the *bla*_OXA-23-like_ gene region between F1 and B1C; (**2**) FITC labeling of the specific probe; (**3**) LAMP amplification using a biotin-labeled FIP primer; (**4**) aliquoting and hybridization of 10 µL of the LAMP product with 1 µL of the FITC-labeled probe for 5 min; and (**5**) detection of the LAMP product using a lateral flow dipstick. The resulting biotin–LAMP product–FITC complex binds to AuNP-conjugated rabbit anti-FITC antibodies and is captured by streptavidin at the test band, producing a red-black band. Free AuNP-conjugated anti-FITC antibodies migrate to the control band and are captured by anti-rabbit IgG, producing a red-black band regardless of the presence of the LAMP product. Absence of the control band indicates an invalid result, likely due to strip degradation [28].

For LAMP-LFD, a biotinylated FIP primer and a 5′-FITC-labeled probe were designed to facilitate specific detection of LAMP amplicons between the B1c and B2 regions (Figure 3). The primer and probe sequences for PCR, LAMP, and LAMP-LFD are listed in Supplemental Table S1 and were synthesized by Bio Basic Inc. (Markham, ON, Canada). 

### 4.5. The Optimization of PCR Conditions

PCR amplification of the *bla*_OXA-23-like_ gene was performed using F3 and B3 primers. Annealing temperature (42–55 °C) and MgCl_2_ concentration (2–6 mM) were optimized using *A. baumannii* AB029 DNA based on clear 195-bp amplification with minimal nonspecific products. PCR was performed in a 25-µL reaction containing 0.16 µM each primer, 200 µM dNTPs, 3 mM MgCl_2_, 1× PCR buffer, 5 U *Taq* DNA polymerase (Vivantis Technologies, Selangor, Malaysia), 1 µL genomic DNA (50 ng), and nuclease-free water. Cycling conditions were 95 °C for 3 min; 30 cycles of 95 °C for 30 s, optimized annealing temperature for 30 s, and 72 °C for 1 min; followed by 72 °C for 6 min. *A. baumannii* AB029 DNA and Milli-Q water served as positive and negative controls, respectively. Amplicons were analyzed by 2% agarose gel electrophoresis at 100 V for 30 min and visualized using a GelDoc Go system (Bio-Rad, Hercules, CA, USA). A 195-bp band matching the positive control was considered positive for *bla*_OXA-23-like_. All PCR reagents were sourced from Vivantis Technologies (Selangor, Malaysia).

### 4.6. The Optimization of LAMP Reaction Conditions

LAMP conditions were optimized using *A. baumannii* AB029 DNA by evaluating amplification temperature (50–70 °C), MgSO_4_ concentration (2.4–6.0 mM), and reaction time (30–60 min), based on clear ladder-like amplification. The 25-µL reaction contained 0.5 µL each of 100 µM FIP/BIP, 0.5 µL each of 10 µM F3/B3, 1.6 µL of dNTPs (25 mM), 2.5 µL of 10× ThermoPol^®^ buffer, 1.3 µL of MgSO_4_ (100 mM), 1.0 µL of *Bst* DNA polymerase, 13.1 µL of sterile distilled water, and 1 µL of genomic DNA (50 ng). Amplification was performed at 63 °C for 45–60 min using a dry bath. *A. baumannii* AB029 DNA and Milli-Q water served as positive and negative controls, respectively. LAMP products were analyzed by 2% agarose gel electrophoresis and visualized using a GelDoc Go system; ladder-like bands indicated a positive result. Carryover contamination was minimized by following standard contamination-control procedures described in the Discussion [37]. Prolonged amplification beyond 60 min may promote nonspecific amplification and false-positive results [38].

LAMP products were analyzed by 2% agarose gel electrophoresis and visualized using a GelDoc Go system; ladder-like bands indicated a positive result. To minimize the carryover, the LAMP assay was conducted according to the standard procedures to minimize carryover, as shown in the discussion [37]. The prolonged amplification time over 60 min can cause nonspecific LAMP products and lead to false positive results [38].

### 4.7. The LAMP–Lateral Flow Dipstick

The development of the LAMP–lateral flow dipstick (LAMP-LFD) for more specific detection of the *bla*_OXA-23-like_ gene involved five steps, as shown in Figure 3 [28].

### 4.8. Determining the Primer Design’s Specificity

Primer specificity was assessed for the LAMP-LFD, LAMP, and PCR assays using 16 non-target bacterial species associated with respiratory, urinary, and bloodstream infections, comprising seven Gram-positive and nine Gram-negative species (Figure 1). Each assay was performed in triplicate.

### 4.9. Determining Analytical Sensitivity

Analytical sensitivity was evaluated by comparing the LAMP-LFD assay with PCR and LAMP for detection of the *bla*_OXA-23-like_ gene in *A. baumannii* AB029. An overnight culture was adjusted to a 0.5 McFarland standard, and DNA was extracted from 1 mL of the suspension. The DNA concentration was measured as 15.74 ng/µL using a NanoDrop™ 2000 spectrophotometer. Ten-fold serial dilutions (15.74 ng/µL to 1.57 fg/µL) were prepared and analyzed in triplicate using PCR, LAMP, and LAMP-LFD assays. The analytical sensitivity also performed in triplicate.

### 4.10. Validity and Predictive Values

LAMP-LFD results were compared with PCR using 2 × 2 contingency tables [43]. Sensitivity, specificity, accuracy, PPV, NPV, LR+, LR−, 95% CI, and Cohen’s kappa (κ) were calculated, with results presented in Supplementary Table S2.

## 5. Conclusions

LAMP-based diagnostic methods reduce equipment requirements and costs while maintaining high analytical performance. Incorporation of specific probes into the LAMP-LFD assay enhances specificity, enabling rapid detection of antimicrobial resistance with visible results within 1–1.5 h, making the assay suitable for resource-limited settings. Future studies should validate this method using multicenter clinical samples from diverse geographic regions, develop multiplex LAMP-LFD platforms for the simultaneous detection of multiple carbapenem resistance genes, and evaluate direct testing of clinical specimens to support rapid CRAB surveillance and outbreak control.

## Figures and Tables

**Table 1:** The results of LAMP and LAMP-LFD for the detection of the *bla*_OXA-23-like_ gene in comparison with the PCR results.

	PCR Positive	PCR Negative	Total
LAMP and LAMP-LFD positive			
Group 1	137	0	137
Group 2	87	0	87
Group 3	0	0	0
True LAMP-LFD positive	224	0	224
LAMP and LAMP-LFD negative			
Group 1	0	33	33
Group 2	0	11	11
Group 3	0	100	100
True LAMP-LFD negative	0	144	144
Total isolates	224	144	368

## Data Availability

The data supporting the findings of this study are available within the article and its Supplementary Materials. Additional data are available from the corresponding author upon reasonable request.

## Supplementary Materials

The following supporting information can be downloaded at https://www.mdpi.com/article/10.3390/ijms27177761/s1.

## Abbreviations

The following abbreviations are used in this manuscript:

°C	degree(s) Celsius
AMR	antimicrobial resistance
ATCC	American Type Culture Collection
CHDLs	Carbapenem-hydrolyzing class D β-lactamases
CRAB	carbapenem-resistant *Acinetobacter baumannii*
CRE	carbapenem-resistant *Enterobacterales*
CRPA	carbapenem-resistant *Pseudomonas aeruginosa*
DNA	deoxyribonucleic acid
fg/μL	femtograms per microliter
HNB	hydroxy naphthol blue
LAMP	loop-mediated isothermal amplification
LFD	lateral flow dipstick
MDR	multidrug-resistant
MgCl_2_	magnesium chloride
MgSO_4_	magnesium sulfate
ng/μL	nanograms per microliter
NPV	negative predictive value
PCR	polymerase chain reaction
PDR	pan-drug-resistant
pg/μL	picograms per microliter
PPV	positive predictive value
UV	ultraviolet
VAP	ventilator-associated pneumonia
WHO	World Health Organization
XDR	extensively drug-resistant
