# Supplementary material for: Development of Loop-Mediated Isothermal Amplification with a Lateral Flow Dipstick for Detection of the BlaOXA-23-like Gene from Carbapenem-Resistant Acinetobacter baumannii
Source: Int J Mol Sci. 2026 Aug 29;27(17):7761. doi: 10.3390/ijms27177761 (PMC13566051; doi:10.3390/ijms27177761)
Supplement: Supplementary file 1 [file ijms-27-07761-s001.zip › ijms-4298676-supplementary.pdf]

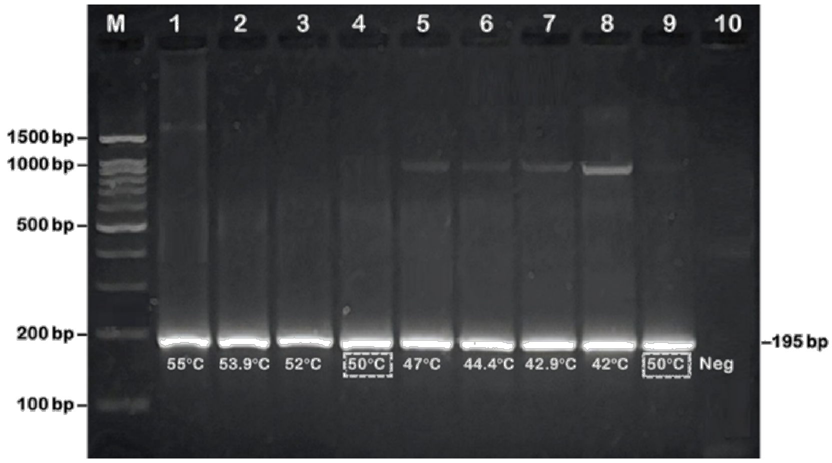

Figure S1. PCR annealing temperature for *bla*$_{OXA-23-like}$ gene detection was optimized between 42 °C and 55 °C. An annealing temperature of 50 °C yielded a specific amplicon without nonspecific bands. Lane M, 100 bp Plus DNA Ladder; lane 10, negative control (Milli-Q water).

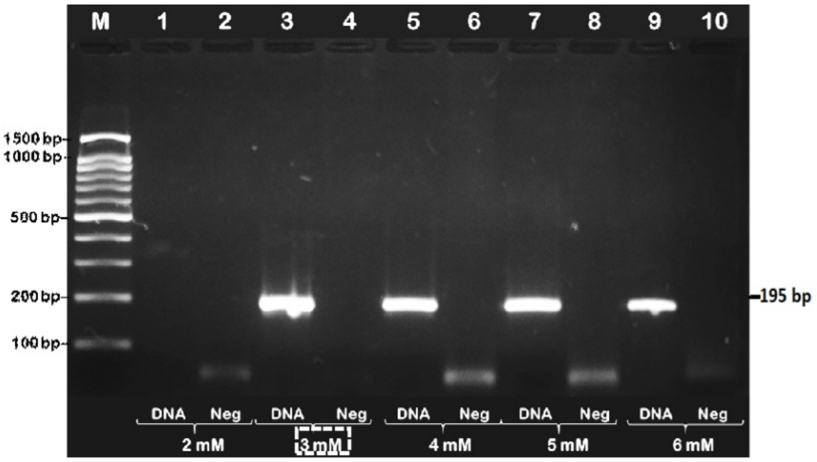

Figure S2. The $MgCl_2$ concentration for PCR detection of *bla*$_{OXA-23-like}$ gene was optimized between 2 and 6 mM, with 3 mM selected as the optimal concentration, yielding a clear, specific amplicon. Lane M, 100 bp Plus DNA Ladder; lane 10, negative control (Milli-Q water).

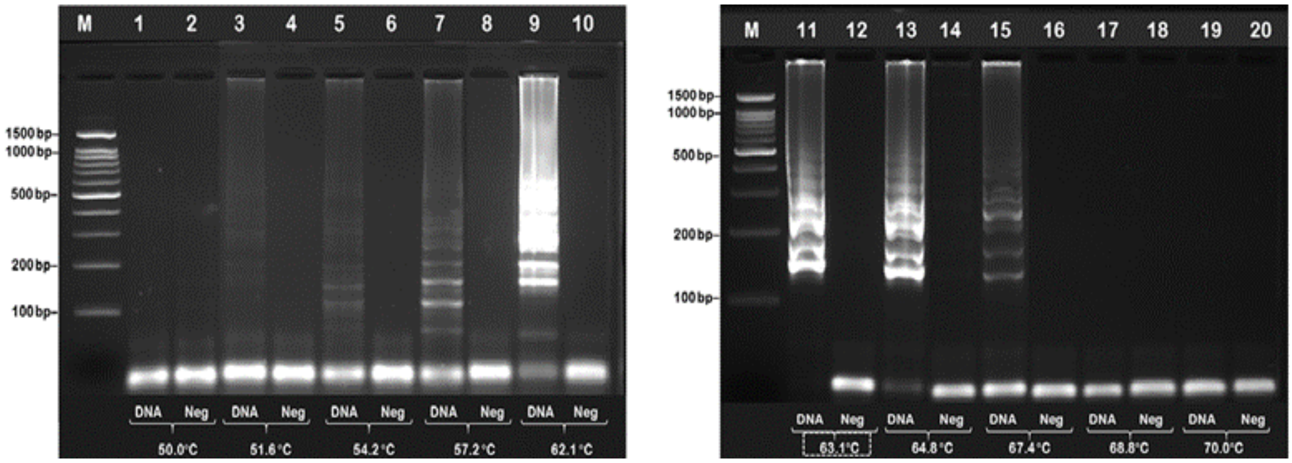

Figure S3. The LAMP amplification temperature for *bla*$_{OXA-23-like}$ gene detection was optimized between 50 °C and 70 °C, with 63 °C selected as the optimal temperature, yielding intense ladder-like bands. Lane M, 100 bp Plus DNA Ladder; lane 10, negative control (Milli-Q water).

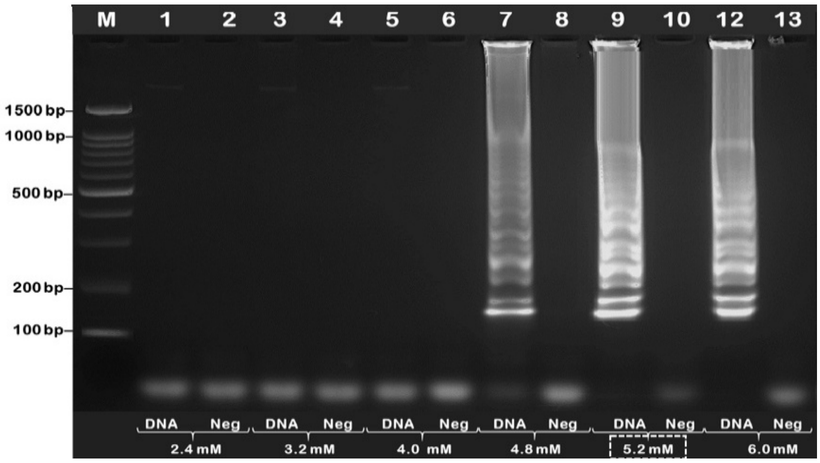

Figure S4. The $MgSO_4$ concentration for LAMP detection of *bla*$_{OXA-23-like}$ gene was optimized between 2.4 and 6.0 mM at 63 °C, with 5.2 mM selected as the optimal concentration, yielding clear ladder-like bands. Lane M, 100 bp Plus DNA Ladder; lane 10, negative control (Milli-Q water).

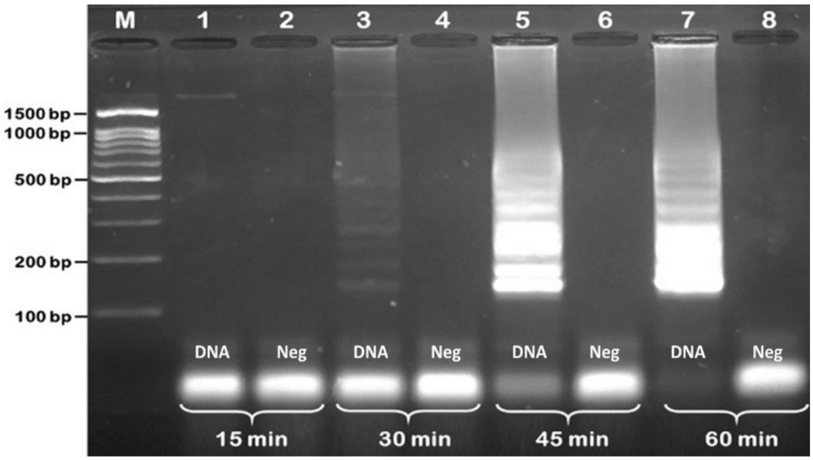

Figure S5. The LAMP amplification time for *bla*$_{OXA-23-like}$ gene detection was optimized at 15, 30, 45, and 60 min. Clear ladder-like bands were observed at 45 and 60 min, and 45 min was selected as the optimal amplification time. Lane M, 100 bp Plus DNA Ladder.

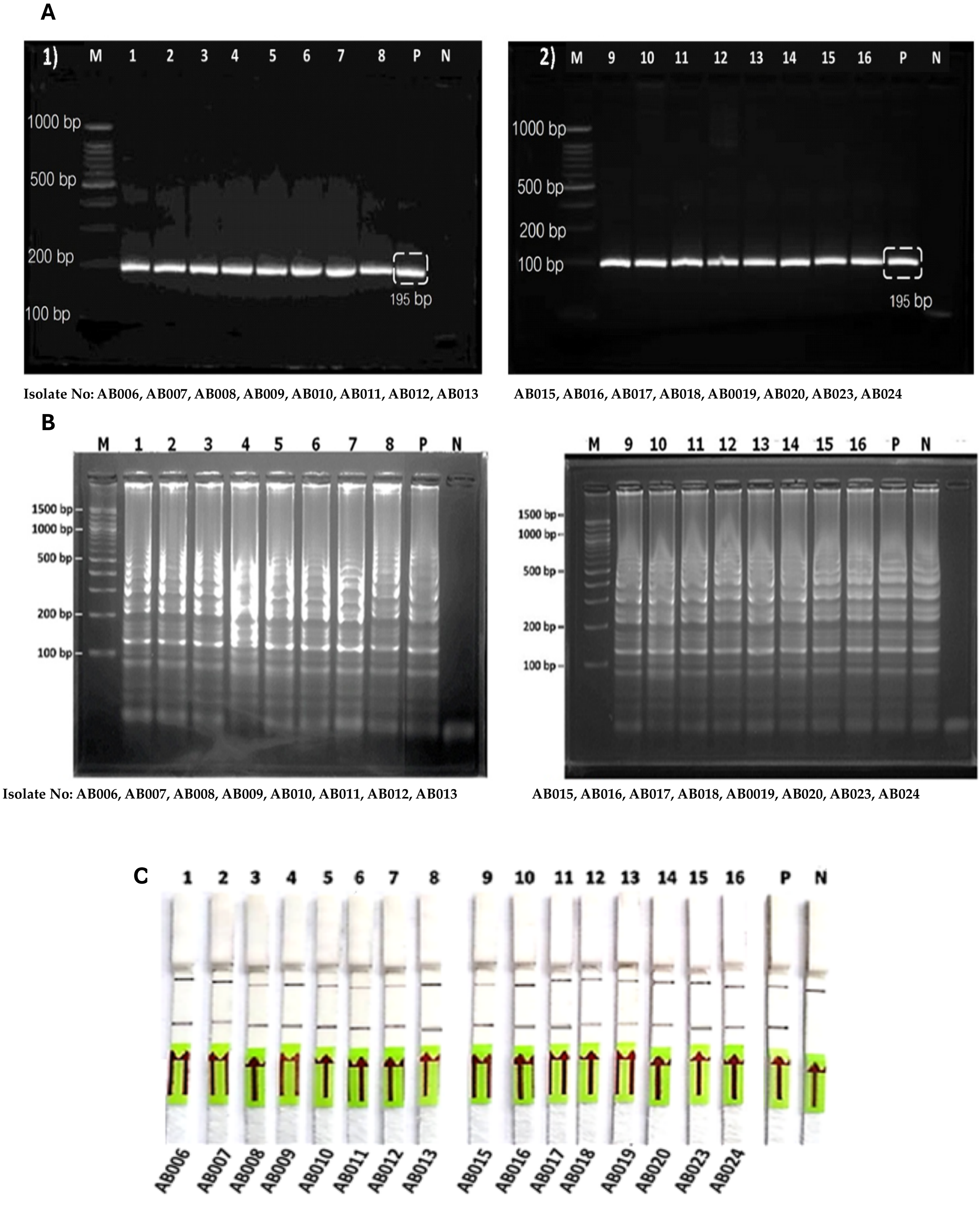

Figure S6. Comparison of (C)LAMP-LFD, (B) LAMP, and (A) PCR assays for 16 of 137 *bla*$_{\text{OXA-23-like}}$ gene- positive CRAB

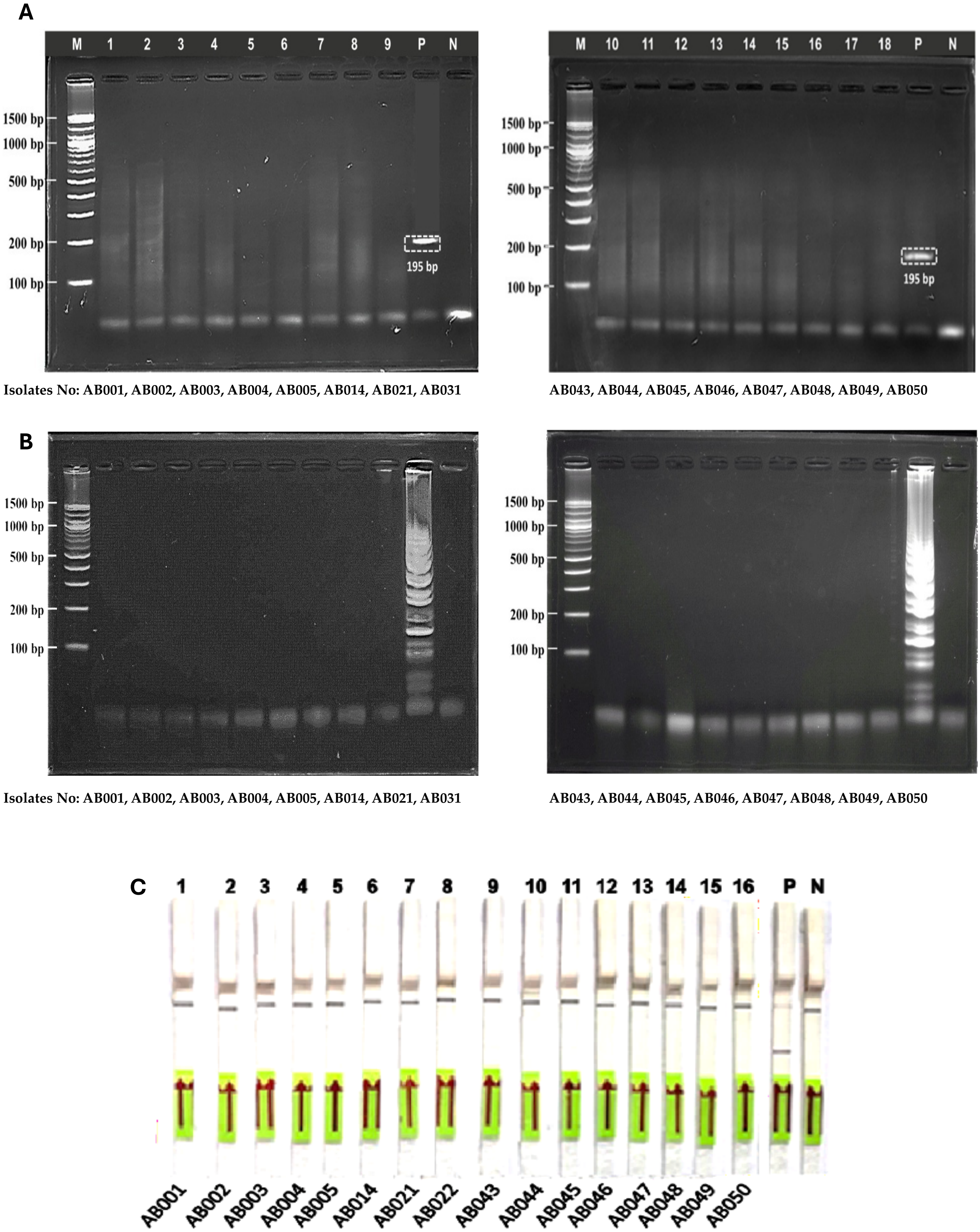

Figure S7. Comparison of LAMP-LFD, LAMP, and PCR assays for 16 of the 33 *bla*$_{OXA-23-like}$ gene-negative CRAB isolates collected in 2018.

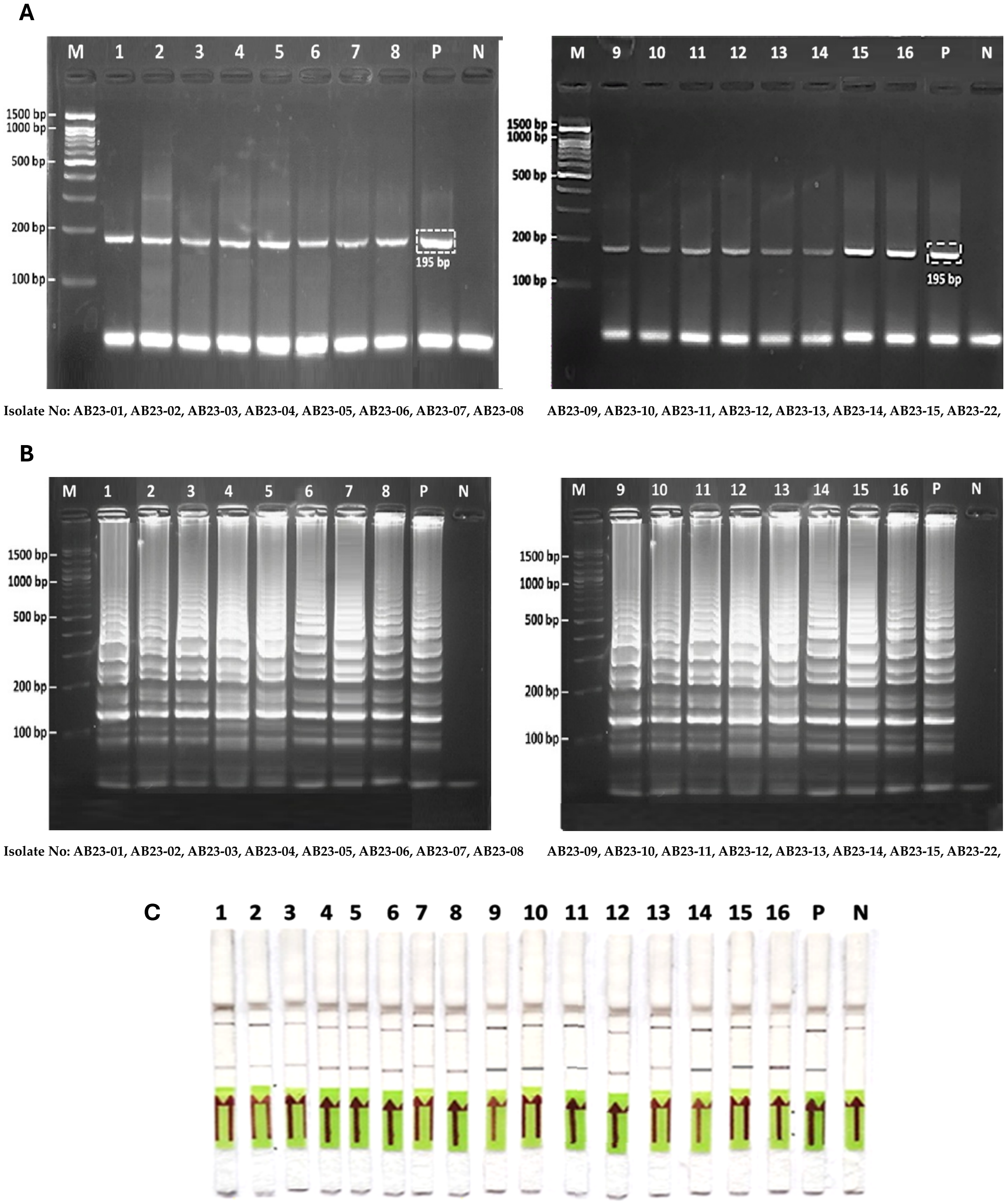

Figure S8. Comparison of (C) LAMP-LFD, (B) LAMP, and (A) PCR assays for 16 of the 87 *bla*$_{\text{OXA-23-like}}$ gene-positive CRAB isolates collected in 2023

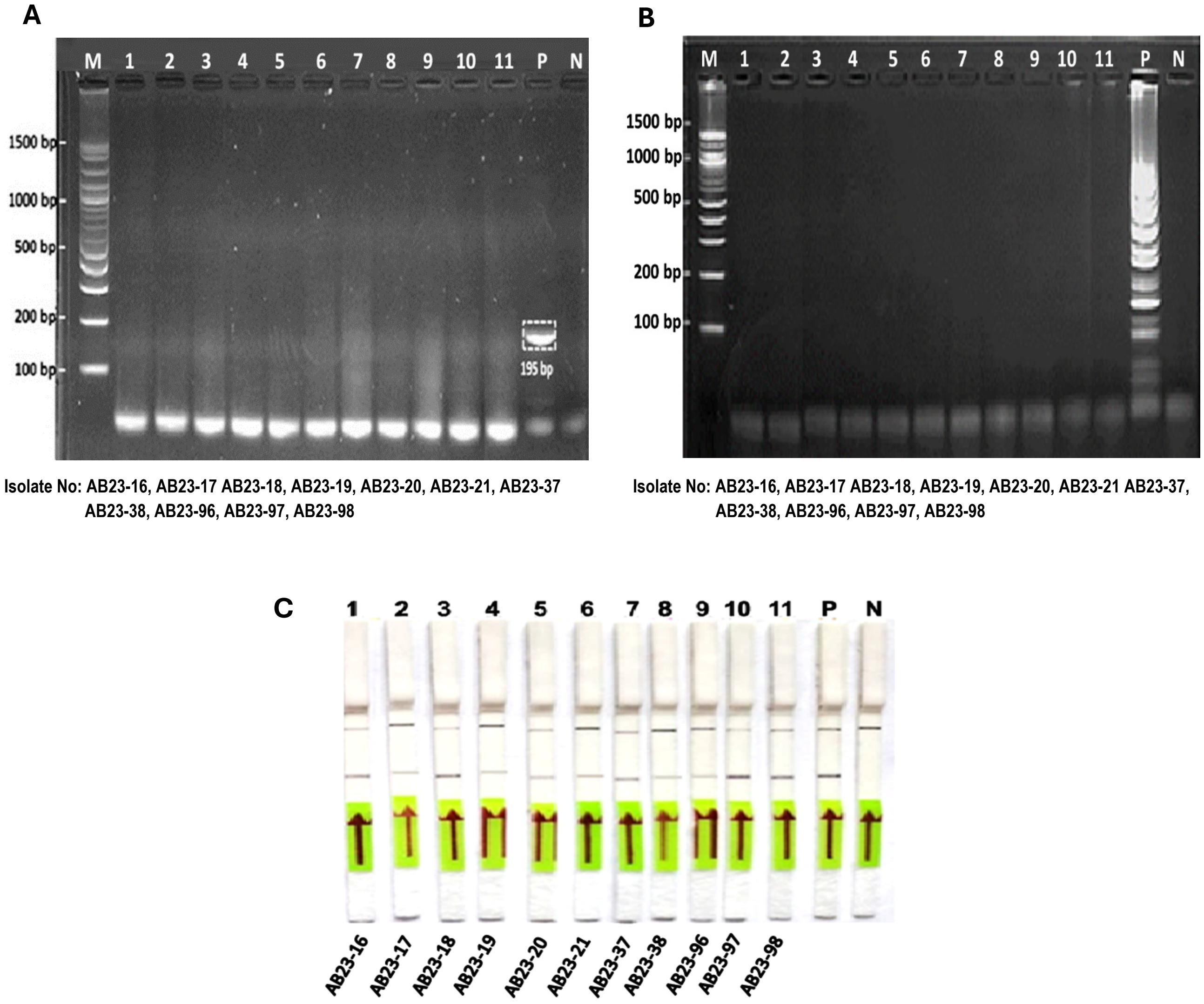

Figure S9. Comparison of (C) LAMP-LFD, (B) LAMP, and (A) PCR assays for all 11 *bla*$_{\text{OXA-23-like}}$ gene-negative CRAB isolates collected in 2023.

A

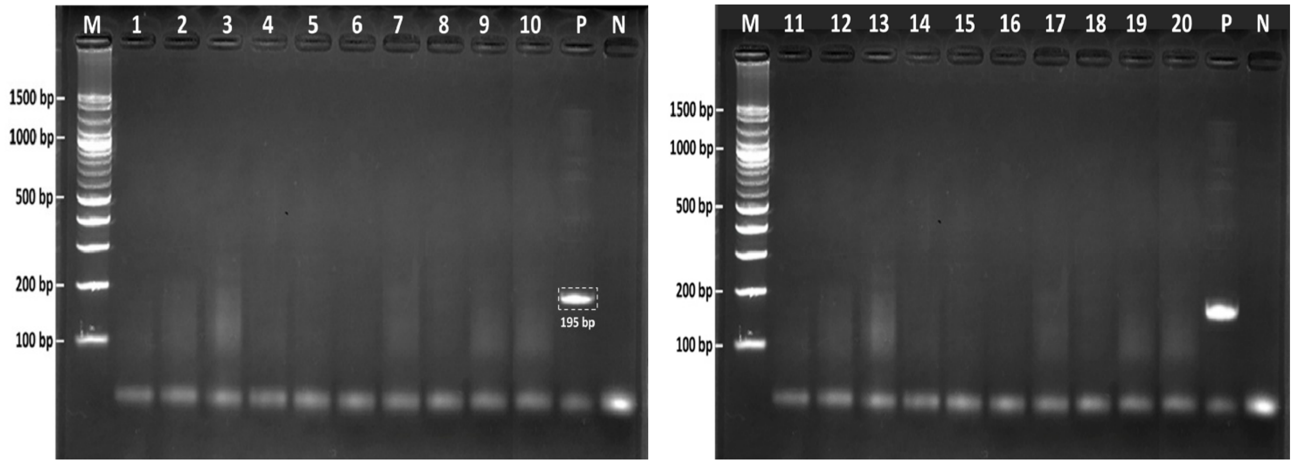

Isolate No: AB18S-1, AB18S-2, AB18S-3, AB18S-4, AB18S-5, AB18S-6, AB18S-7, AB18S-8

Isolate No: AB23S-1, AB23S-2, AB23S-3, AB23S-4, AB23S-5, AB23S-6, AB23S-7, AB23S-8

B

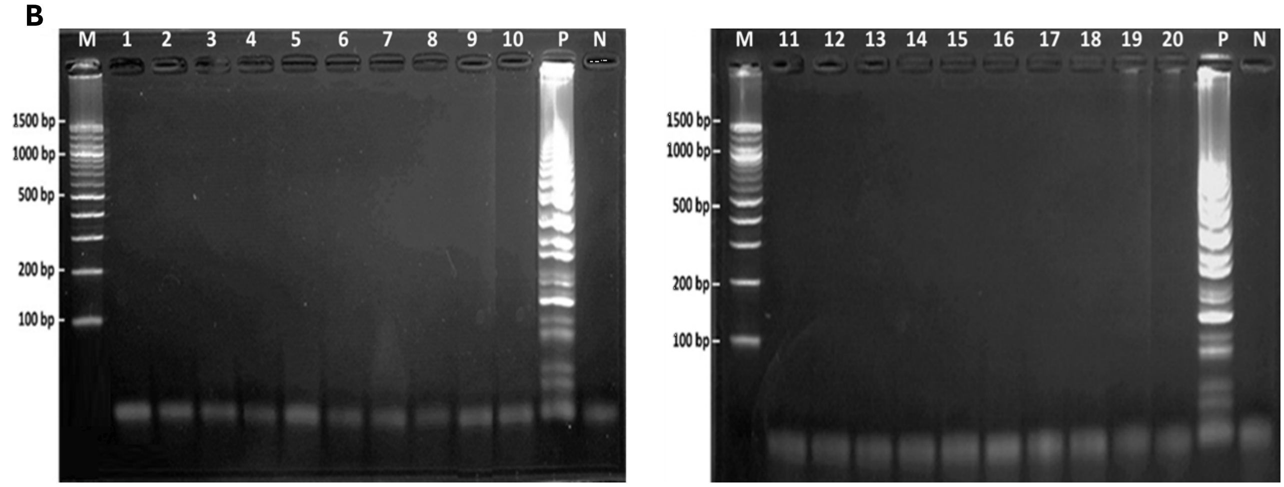

Isolate No: AB18S-1, AB18S-2, AB18S-3, AB18S-4, AB18S-5, AB18S-6, AB18S-7, AB18S-8

Isolate No: AB23S-1, AB23S-2, AB23S-3, AB23S-4, AB23S-5, AB23S-6, AB23S-7, AB23S-8

C

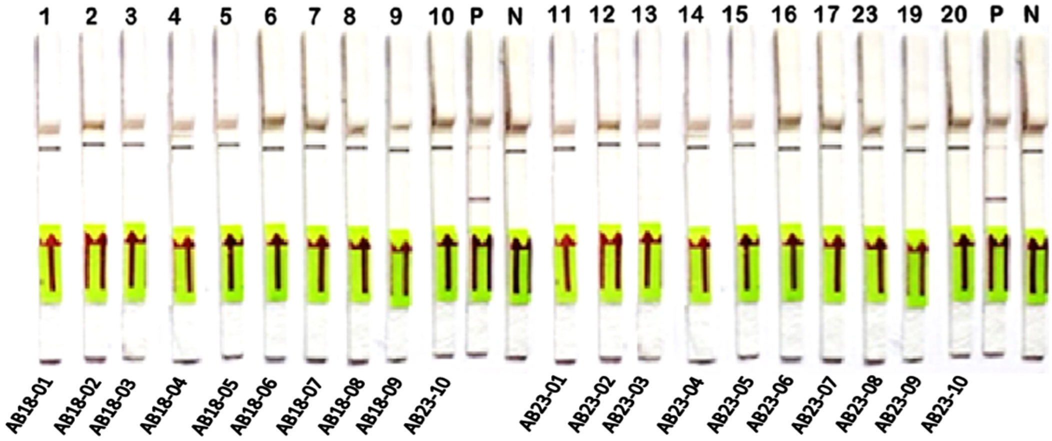

Figure S10. Representative comparison of (C) LAMP-LFD, (B) LAMP, and (A) PCR assays for *bla*$_{\text{OXA-23-like}}$ gene detection in 10 of the 50 carbapenem-susceptible *A. baumannii* isolates collected in 2018 and 10 of the 50 collected in 2023, all of which were negative.

Table S1. The calculation of %Specificity, %Sensitivity, Positive predictive value (PPV), Negative predictive value (NPV), Positive likelihood ratio, Negative likelihood ratio, and 95% confidence interval

Statistical Analysis	Formula	Results
**Sensitivity**	TP/TP+FN	100%
**Specificity**	TN/TN+FP	100%
**Accuracy**	TP+TN/TP+FP+TN+FN	100%
**Positive predictive value (PPV)**	TP/TP+FP	100%
**Negative predictive value (NPV)**	TN/TN+FN	100%
**Positive likelihood ratio (LR⁺)**	Sensitivity/1-Specificity	∞
**Negative likelihood ratio (LR-)**	1-Sensitivity/Specificity	0
**95% confidence interval (CI)**	$\overline{x} \pm z\frac{s}{\sqrt{n}}$	79.1% - 88.0%
**Cohen's Kappa (κ)**	$\frac{2 \times (TP \times TN - FN \times FP)}{(TP+FP) \times (FP+TN) + (TP+FN) \times (FN+TN)}$	1

When:	True Positive (TP)	= 224
	False Positive (TP)	= 0
	True Negative (TP)	= 44
	False Negative (TN)	= 0
	Sample mean	= 0.84
	Sample standard deviation	= 0.37
	z-value for 95% confidence	= 1.96

Table S2. The results of LAMP and LAMP-LFD for the detection of the *bla*$_{\text{OXA-23-like}}$ gene.

Assays	Primer	DNA Sequences 5′ to 3′	Amplicon
**PCR**	Forward primer-OXA-23 (F3)	5' TGT CTT GTT CAA TTA ACC ATG T 3'	195 bp
	Reverse primer- OXA-23 (B3)	5' GCA ATC GTT TAT CGT GGA AT 3'	
**LAMP**	F3 primer OXA-23	5' TGT CTT GTT CAA TTA ACC ATG T 3'	Ladder-like bands
	B3 primer OXA-23	5' GCA ATC GTT TAT CGT GGA AT 3'	
	FIP primer OXA-23	5' ATA GCC ATT TTT GGT AAA GAC CGT TTT TTG CTC ACC GAT AAA CTC TCT G 3'	
	BIP primer-OXA-23	5' ATA GCC ATT TTT GGT AAA GAC CGT TTT TTG CTC ACC GAT AAA CTC TCT G 3'	
**LAMP-LFD**	F3 primer OXA-23	5' TGT CTT GTT CAA TTA ACC ATG T 3'	
	B3 primer OXA-23	5' GCA ATC GTT TAT CGT GGA AT 3'	
	Biotin-FIP primer OXA-23	5' biotin-ATA GCC ATT TTT GGT AAA GAC CGT TTT TTG CTC ACC GAT AAA CTC TCT G 3'	
	BIP primer	5' ATA GCC ATT TTT GGT AAA GAC CGT TTT TTG CTC ACC GAT AAA CTC TCT G 3'	
**Specific Probe**	FITC-Probe- OXA-23	5′ FITC-TTG TGA ATG TGT TCG CAG 3′	
